# Incidence and Mortality Patterns of Acute Myeloid Leukemia in Belgrade, Serbia (1999–2013)

**DOI:** 10.3390/medicina54010005

**Published:** 2018-03-20

**Authors:** Natasa Maksimovic, Milica Zaric, Tatjana Gazibara, Goran Trajkovic, Gorica Maric, Dragan Miljus, Nada Suvajdzic Vukovic, Dragica Tomin, Marijana Virijevic, Darija Kisic Tepavcevic, Tatjana Pekmezovic

**Affiliations:** 1 Institute of Epidemiology, Faculty of Medicine, University of Belgrade, 11000 Belgrade, Serbia; natamax07@yahoo.com (N.M.); milicamzaric@yahoo.com (M.Z.); gazibara@drenik.net (T.G.); goricamaric87@gmail.com (G.M.); darijakt@gmail.com (D.K.T.); 2 Institute of Medical Statistics and Informatics, Faculty of Medicine, University of Belgrade, 11000 Belgrade, Serbia; gtrajkovic@gmail.com; 3 Institute of Public Health of Serbia “Dr Milan Jovanovic Batut”, Dr Subotica 5, 11000 Belgrade, Serbia; dragan_miljus@batut.org.rs; 4 Clinic for Hematology, Clinical Center of Serbia, 11000 Belgrade, Serbia; Suvajdzic.nada@gmail.com (N.S.V.); dragica.tomin@med.bg.ac.rs (D.T.); marijana.virijevic@yahoo.com (M.V.)

**Keywords:** acute myeloid leukemia, incidence, mortality, trend

## Abstract

*Introduction*: To assess incidence and mortality trends of acute myeloid leukemia (AML) in Belgrade (Serbia) in a 15-year period (from 1999 to 2013). *Material and Methods:* Data were obtained from the Cancer Registry of Serbia, Institute of Public Health of Serbia. Standardized incidence and mortality rates per 100,000 inhabitants were calculated by direct standardization method using World Standard Population. Analysis of raw data indicated single-digit numbers per year and per 5-year age cohorts. Therefore, we merged years of diagnosis to three-year intervals, creating so-called “moving averages”. We also merged study population to 10-year age cohorts. *Results:* Both incidence and mortality rates increased with age, i.e., the lowest rates were observed in the youngest age groups and the highest rates were observed in oldest age groups. In all age groups, except the youngest (15–24 years), AML incidence was statistically significantly higher in men compared with women. Average age-adjusted incidence was 2.73/100,000 (95% confidence interval (CI) 2.28–3.71). Average age-adjusted mortality was 1.81/100,000 (95% CI 1.30–2.26). Overall, there were no significant changes in incidence trend. Age-adjusted incidence rates had increasing tendency among men aged 65–74 years (B = 0.80, standard error (SE) = 0.11; *p* = 0.005) and in total population aged 65–74 years (B = 0.41, SE = 0.09; *p* = 0.023). Increasing tendency in incidence of AML among women was observed in age group >75 years (B = 0.63, SE = 0.14; *p* = 0.019). No changes of mortality trend were observed. *Conclusion:* There was no significant change in trends of AML from 1999 to 2013 in the population of Belgrade.

## 1. Introduction

Acute myeloid leukemia (AML), a cancer of myeloid cells in the bone marrow, is the most common acute leukemia in the adult population [1,2]. Because AML incidence rises with age [1,2], this condition is increasingly relevant as the population at a global level is aging. Median age at diagnosis of AML is approximately 67 years [3]. More than one half of persons diagnosed with AML are older than 65 years and approximately one third are older than 75 years [4]. By contrast, occurrence of AML is less common in persons younger than 45 years [5]. Additionally, there are specific geographic variations in AML incidence [6]. Namely, North America, Western Europe and Australia report the highest rates of AML, while in Asia and Latin America AML appears to occur less frequently [3,6,7]. Some evidence suggests that discrepancies in AML occurrence result from inaccurate and inconsistent reporting of AML cases [3,6,7].

Acute myeloid leukemia accounts for approximately one third of all leukemias among adults in developing countries [3]. In the period 2009–2013, AML age-adjusted incidence rate of 4.1 per 100,000 persons was reported in the United States. In Europe, estimated incidence ranged from 5 to 8 per 100,000 for the same period [5]. Reports of GLOBOCAN suggest that age-standardized rate of AML for 2012 was 3.8 per 100,000 in middle-income countries and 2.5 per 100,000 individuals in countries categorized as “low-income” [8].

Prognosis of AML, particularly among older persons, remains poor despite advances in diagnostic and therapeutic modalities [9]. The age-adjusted mortality rate of AML in the United States was estimated at 2.8 per 100,000 in the period 2009–2013 [3], while annual mortality rate in Europe varied between 4 to 6 per 100,000 [5]. According to GLOBOCAN in 2012, estimated age-standardized mortality rate of AML was 3.2 per 100,000 persons in middle-income countries, whereas incidence in low-income countries accounted for 2.4 per 100,000 [8].

Studies on temporal trends of incidence and mortality of AML are inconsistent. In fact, the incidence and mortality of AML in the Balkan region remain understudied. Given that incidence and mortality rates represent indicators and potential for prevention, quantification of risk of developing and dying from AML and their changes over time could offer valuable insight into the dynamics of AML. The aim of this study was to assess incidence and mortality trends of AML in Belgrade (Serbia) in a 15-year period (from 1999 to 2013).

## 2. Materials and Methods

In this descriptive study AML was diagnosed according to the World Health Organization (WHO) classifications and defined as ICD-O-3 and ICD10 code C92.0 [10,11]. Those AML developing from myelodysplastic syndrome or myeloproliferative neoplasms or secondary to chemotherapy/radiation were also recorded. Incidence data for the period 1999–2013 were obtained from the Cancer Registry of Serbia, Institute of Public Health of Serbia [12]. The number of AML deaths in the Belgrade region, as well as the population data for the same time period, was provided by the Statistical Office in Belgrade.

### Data Analysis

We used specific and standardized incidence and mortality rates to describe the risk of developing and dying from AML. Standardized incidence and mortality rates per 100,000 inhabitants were calculated by direct standardization method, using the World Standard Population proposed by Segi [13]. Confidence intervals (CI) for incidence and mortality rates were calculated at the probability level of 95% [14]. Probability level of *p* < 0.05 was considered statistically significant.

When calculating AML incidence and mortality trends, we did not use per-year rates. Initial analysis of raw data showed single-digit numbers per year and per 5-year age cohorts. Thus, we merged years of diagnosis to three-year intervals and modified the study population to 10-year age cohorts instead. By doing this, i.e., creating so-called “moving averages”, we aimed at achieving more statistical power and robustness of the regression analysis. Analysis of incidence and mortality trends using per-year rates and 5-year age cohorts indicated unrealistic rises and drops in incidence and mortality that did not reflect an objective epidemiological situation, but a statistical error. By merging years of diagnosis and age cohorts, we obtained more stable data and, thus, reduced probability of statistical error. Specific incidence and mortality rates were calculated for both genders and for the following age groups: 15–24, 25–34, 35–44, 45–54, 55–64, 65–74 and >75 years.

## 3. Results

During the observed period, a total of 1018 new AML cases and 759 deaths due to AML were registered in the population of Belgrade (on average 68 new cases and 51 deaths annually). Male-to-female ratios were 1.6 and 1.4 for incidence and mortality, respectively.

Average age-specific and age-adjusted incidence and mortality rates from AML for the total population are presented in Table 1 and Table 2. Both incidence and mortality rates increased with age, i.e., the lowest rates were observed in the youngest age groups, and the highest rates were observed in the older age groups. Furthermore, in all age groups, except the youngest, 15–24 years, AML incidence was statistically significantly higher in men compared with women (Table 3). In terms of mortality, overall mortality rate was statistically significantly higher in men compared to women, as well as in the following age groups: 25–24, 55–64 and 65–74 years (Table 4). In the investigated time period, the average age-adjusted incidence rates from AML were 3.70/100,000 (95% CI 3.00–4.44) among men, 1.88/100,000 (95% CI 1.18–3.46) among women and 2.73/100,000 (95% CI 2.28–3.71) in the total population. Average age-adjusted mortality rates from AML in the same period were 2.57/100,000 (95% CI 1.53–4.04) among men, 1.35/100,000 (95% CI 1.01–1.85) among women and 1.81/100,000 (95% CI 1.30–2.26) for the total population.

Table 5 and Table 6 display data on incidence and mortality trends from AML. Overall, there were no significant changes in incidence and mortality trends among men, women and in the total population. Age-adjusted incidence rates had increasing tendency among men aged 65–74 years (B = 0.80, standard error (SE) = 0.11; *p* = 0.005) and in total population (B = 0.41, SE = 0.09; *p* = 0.023) of that same age. Increasing tendency in incidence of AML among women was observed in age group >75 years (B = 0.63, SE = 0.14; *p* = 0.019) (Table 5).

When trends of mortality rates were analyzed, we observed that changes in trend were not present in all three investigated groups (men, women or total population) (Table 6).

## 4. Discussion

Based on the results of our analysis, we report that increasing tendency in incidence and mortality trends from AML were observed during 1999–2013; however, this was not statistically significant. Estimated AML incidence in the Belgrade population accounted for 3.7 per 100,000 among men and 1.88 per 100,000 among women. In consideration of the risk of developing AML in the Balkan region, such as in neighboring Croatia, age-standardized rates for men were 1.41 per 100,000 persons in the period 1988–1992 and increased to 2.30 per 100,000 persons in the period 2005–2009, which is approximately a 63% change [15]. A similar pattern was observed in the female population, with an age-standardized rate of AML of 1.06 per 100,000 persons between 1988 and 1992 that rose to 1.63 per 100,000 persons in the period 2005–2009 [15], which matches our findings.

In other European countries, such as Denmark, incidence rates from 1980 to 2010 were approximately 15–20 per 100,000 person-years in the older population, compared to only 2–3 per 100,000 person-years among younger persons [16]. Overall, in Western countries, age-standardized incidence rate is around 3–4 per 100,000 persons [17,18]. In other regions, such as Brazil, a decrease in AML mortality was reported [19]. In fact, that analysis showed changes over time (1994–2011), where drops in mortality were reported in the first half, while there was an increase in the following 3 years among both men and women [19]. Due to a likely increase in life expectancy in forthcoming decades and aging of the population in Serbia, there is a shift favoring older population groups. Therefore, incidence of AML could have a rising trend following the changes in age structure of the population. However, better access to improved and more potent therapies free of charge could, in fact, contribute to a decrease in mortality.

Although occurrence of AML was reported in all age groups, it is well established that AML incidence rises with age, and therefore, accounts for approximately 25% of all leukemias diagnosed in adults [20]. In particular, frequent occurrence was observed among persons in the age range 65 to 74 years [21]. In the US, AML represents 1.2% of all newly diagnosed malignant diseases. Incidence rate in the population older than 70 years of age reaches 15–25 per 100,000 [5]. In the period 2011–2013 in the UK, almost 6 out of 10 persons (55%) diagnosed with AML were older than 70 years [22]. Furthermore, the increase of AML incidence rates in almost all age groups in the UK has been observed since the end of the 1970s [22]. Thus, the increase of age-standardized incidence rates of more than 100% in the period 1979–1981 and 2011–2013 among people older than 80 years seems expected. In the same period, incidence has been rising for only one third in people aged 50–59, while in those aged 25–49 the rates remained stable [21]. Despite the fact that incidence rates in different age groups in the US remained stable over time, over the past 10 years a slight annual increase of about 3.4% was observed among the oldest [3].

We observed a significant increase in mortality in age group 65–74 years in both males and total population and among females aged >75 years. Similar to our study, the percent of AML deaths in the US population was the highest among people aged 75–84 years [3]. Also, like mortality rates in the Belgrade population, mortality rates in the United States remained stable in the period 2004–2013 [3]. Age at diagnosis seems to be one of the most important prognostic features of AML, with prognosis being poorer at older age, even when accounting for cytogenetic risk groups [23]. Studies exploring the association between age and AML outcomes suggested that the prognosis was poorer in persons older than 60–65 years of age [24]. These findings could be explained by characteristics of persons (e.g., worse health status among older persons, more comorbidities and poor tolerance for intensive chemotherapy), as well as by the very biology of illness (e.g., an increased incidence of high-risk chromosomal abnormalities and higher frequency of multi-drug resistance expression) [25]. In terms of the Belgrade population, given the increasing AML mortality, we hypothesize that it could be a result of diminished access to the effective treatment. Namely, allogenic bone marrow transplantation from a matched unrelated donor has not been available in Serbia before January 2013 and has been restricted to persons younger than 60 years of age. Beside this, hypomethylating agents were not available for the treatment of the elderly frail AML patients, while arsenic trioxide has not yet been available for treatment of the acute promyelocytic leukemia.

In the present study, incidence and mortality rates were statistically significantly higher among men compared to women in all age groups. According to data from Cancer Research United Kingdom [22], progressive increase of age-specific AML incidence rates was observed in age range 40–44 years. This increase was more prominent among persons aged 60–64 years, while incidence rates were the highest among men aged 85–89 years and women aged above 90 years. Furthermore, that study found a difference in incidence rates between men and women aged 35–39 and over 50 years, with higher incidence in men, while other age groups did not differ amongst each other [22]. However, difference in incidence is especially pronounced in age range 35–39 years, with a male-to-female ratio of 18:10. The same report suggested that the number of new cases of AML rose by 61% from 1979–1981 to 2011–2013 among females [22]. Over the past decade, AML age-adjusted incidence rates have increased by 7% in the United Kingdom, including an 8% increase among men, while rates remained stable among women [22]. A potential reason for this increase in AML incidence trends appears to be better diagnostic techniques and more precise updating of the cancer register [26].

There are several limitations of our study that need to be addressed. Potential limitations are related to the reliability, completeness, quality and accuracy of death certifications for cancers and underreporting, which may vary across calendar years. Due to registry drawbacks, it is possible that some incident cases were omitted. Also, there might be time differences in quality and utilization of medical care, particularly at the beginning of the observed period, specifically, because of the NATO bombing of the former Yugoslavia in 1999 and political changes from a socialist to democratic government in the years 2000–2002. Similarly, we should also account for differences in diagnostic criteria. Specifically, up to 2008 AML was diagnosed by marrow blasts ≥30%, whereas the WHO classification changed the criteria in 2008 to 20%. Therefore, persons with 20% blast count have been omitted from the register in the period 1999–2008. Since only data from 1999 to 2013 were available, it was not possible to estimate AML incidence and mortality trends over a longer time period. Additionally, AML includes several subtypes, different in terms of treatment and prognosis, which could have affected our mortality data. Complete remission of AML occurs in less than one half of persons younger than 60 years of age and in less than 15% of persons above 60 years of age [27].

## 5. Conclusions

In summary, although increasing tendency in incidence and mortality rates from AML were noted, there is no significant change in trend in AML from 1999 to 2013 in the Belgrade population. However, a significant increase in mortality was observed among men and in the total population aged 65–74 years and women aged >75 years. Both incidence and mortality rates were rising with age, which is in accordance with literature data. Our study suggests that there is a need for improvement and timely introduction of optimal therapeutic choices covered by health insurance. Future research should include estimates of AML incidence and mortality for the entire population of the country.

## Figures and Tables

**Table 1:** Average age-specific and age-adjusted incidence rates of acute myeloid leukemia in Belgrade population, 1999–2013.

Age Group (Years)	Men	Women	Total
	Mean (95% CI)	Mean (95% CI)	Mean (95% CI)
15–24	1.90 (0.00–3.70)	1.38 (0.00–3.33)	1.64 (0.47–3.28)
25–34	2.64 (0.79–5.69)	0.81 (0.00–2.67)	1.70 (0.77–2.76)
35–44	3.84 (1.00–6.97)	1.69 (0.00–5.34)	2.71 (0.47–5.64)
45–54	6.36 (3.19–8.64)	3.05 (0.85–6.97)	4.60 (2.60–7.44)
55–64	9.78 (3.65–19.48)	5.28 (1.04–10.44)	7.35 (3.37–14.05)
65–74	14.94 (7.67–18.81)	7.84 (4.26–14.90)	10.97 (5.99–16.03)
75+	17.01 (3.31–36.41)	9.11 (2.30–16.52)	12.16 (3.82–19.08)
Age-adjusted incidence rates	3.70 (3.00–4.44)	1.88 (1.18–3.46)	2.73 (2.28–3.71)

CI—confidence interval.

**Table 2:** Average age-specific and age-adjusted mortality rates of acute myeloid leukemia in Belgrade population, 1999–2013.

Age Group (Years)	Men	Women	Total
	Mean (95% CI)	Mean (95% CI)	Mean (95% CI)
15–24	0.45 (0.00–1.85)	0.40 (0.00–1.89)	0.45 (0.00–1.40)
25–34	1.11 (0.00–2.85)	0.58 (0.00–1.78)	0.84 (0.00–1.84)
35–44	2.45 (0.00–8.21)	0.94 (0.00–2.67)	1.40 (0.47–2.56)
45–54	3.27 (0.00–7.99)	1.88 (0.00–4.18)	2.38 (0.74–5.21)
55–64	7.09 (3.65–14.61)	4.12 (1.04–7.30)	5.37 (2.25–8.43)
65–74	14.99 (8.06–24.19)	7.18 (3.19–10.64)	10.42 (5.34–14.85)
75+	18.18 (3.31–46.34)	9.59 (4.13–14.89)	11.79 (3.82–20.35)
Age-adjusted mortality rates	2.57 (1.53–4.04)	1.35 (1.01–1.85)	1.81 (1.30–2.16)

CI—confidence interval.

**Table 3:** Differences in acute myeloid leukemia (AML) incidence between genders: results of regression analysis.

Age Group (Years)	Male vs. Female		
	B Coefficient	SE	*p*
15–24	−0.52	0.40	0.229
25–34	−1.83	0.45	**0.005**
35–44	−2.14	0.37	**0.001**
45–54	−3.31	0.53	**<0.001**
55–64	−4.50	1.24	**0.008**
65–74	−7.71	1.75	**0.005**
75+	−7.90	2.10	**0.007**
Age-adjusted incidence rates	−1.18	0.18	**<0.001**

SE—standard error; *p*—probability level. Bold values denote statistical significance.

**Table 4:** Differences in AML mortality between genders: results of regression analysis.

Age Group (Years)	Male vs. Female		
	B Coefficient	SE	*p*
15–24	−0.06	0.21	0.770
25–34	−0.52	0.19	**0.028**
35–44	−1.09	0.64	0.135
45–54	−1.23	0.71	0.128
55–64	−2.46	0.95	**0.036**
65–74	−0.00	0.00	**0.002**
75+	−8.58	4.13	0.076
Age-adjusted mortality rates	−1.22	0.86	**0.002**

SE—standard error; *p*—probability level. Bold values denote statistical significance.

**Table 5:** Trends of incidence rates from AML, in Belgrade population, 1999–2013.

Age Group (Years)	Men			Women			Total		
	B	SE	*p*	B	SE	*p*	B	SE	*p*
15–24	−0.01	0.08	0.316	0.02	0.04	0.683	−0.04	0.05	0.463
25–34	−0.02	0.09	0.860	−0.03	0.07	0.734	−0.21	0.03	0.521
35–44	−0.015	0.05	0.062	0.03	0.03	0.394	−0.05	0.04	0.247
45–54	0.12	0.05	0.115	−0.01	0.11	0.923	0.05	0.06	0.513
55–64	0.28	0.26	0.331	−0.04	0.14	0.811	0.11	0.18	0.587
65–74	0.02	0.25	0.929	−0.08	0.36	0.833	−0.04	0.31	0.907
75+	0.41	0.49	0.461	−0.06	0.08	0.506	0.12	0.17	0.515
Age-adjusted incidence rates	<0.001	0.03	1.000	−0.00	0.03	0.858	−0.00	0.03	0.958

SE—standard error.

**Table 6:** Trends of mortality rates from AML, in Belgrade population, 1999–2013.

Age Group (Years)	Men			Women			Total		
	B	SE	*p*	B	SE	*p*	B	SE	*p*
15–24	0.02	0.05	0.760	0.01	0.02	0.401	0.02	0.02	0.330
25–34	−0.03	0.02	0.216	−0.02	0.04	0.636	−0.03	0.03	0.402
35–44	0.13	0.10	0.280	−0.03	0.12	0.798	0.04	0.02	0.251
45–54	−0.04	0.12	0.780	−0.16	0.13	0.306	−0.13	0.09	0.276
55–64	−0.00	0.19	0.989	−0.08	0.14	0.652	−0.06	0.10	0.568
65–74	0.80	0.11	**0.005**	0.16	0.23	0.543	0.41	0.09	**0.023**
75+	1.83	0.92	0.141	0.63	0.14	**0.019**	0.79	0.46	0.183
Age-adjusted mortality rates	0.09	0.05	0.165	−0.01	0.01	0.617	0.02	0.02	0.358

SE—standard error; Bold values denote statistical significance.
